# miR-27b inhibits gastric cancer metastasis by targeting NR2F2

**DOI:** 10.1007/s13238-016-0340-z

**Published:** 2016-11-14

**Authors:** Qingzhao Feng, Xionglin Wu, Fuchao Li, Beibei Ning, Xiaofeng Lu, Yin Zhang, Ying Pan, Wenxian Guan

**Affiliations:** 1 Department of General Surgery, The Affiliated Drum Tower Hospital of Medical School of Nanjing University, Nanjing, 210008 China; 2 Department of Gerontology, The Affiliated Drum Tower Hospital of Medical School of Nanjing University, Nanjing, 210008 China; 3 Department of Orthopaedics, The Affiliated Drum Tower Hospital of Medical School of Nanjing University, Nanjing, 210008 China; 4 The Children’s Hospital of Zhejiang University School of Medicine, Hangzhou, 310003 China; 5 Department of Emergency, The Yiwu Affiliated Hospital of Wenzhou Medical University, Yiwu, 322000 China

**Keywords:** miR-27b, NR2F2, gastric cancer, tumor metastasis

## Abstract

Increasing attention is focused on the down-regulation of miRNAs in cancer process. Nuclear receptor subfamily 2 (NR2F2, also known as COUP-TFII) is involved in the development of many types of cancers, but its role in gastric cancer remains elusive. In this experiment, oncomine and Kaplan-meier database revealed that NR2F2 was up-regulated in gastric cancer and that the high NR2F2 expression contributed to poor survival. MicroRNA-27b was targeted and down-regulated by NR2F2 in human gastric cancer tissues and cells. The ectopic expression of miR-27b inhibited gastric cancer cell proliferation and tumor growth *in vitro* and *in vivo*. Assays suggested that the overexpression of miR-27b could promote MGC-803 cells’ migration and invasion and retard their metastasis to the liver. In addition, down-regulation of miR-27b enhanced GES-1 cells’ proliferation and metastasis *in vitro*. These findings reveal that miR-27b is a tumor suppressor in gastric cancer and a biomarker for improving patients’ survival.

**Electronic supplementary material:**

The online version of this article (doi:10.1007/s13238-016-0340-z) contains supplementary material, which is available to authorized users.

## INTRODUCTION

Gastric cancer (GC), the second leading cause of cancer-induced death, has a high incidence in countries like China or Japan (Kang et al., 2014; Yan et al., 2015). Various innate and acquired factors, including *H. pylori* infection, genetic, epigenetic and molecular alterations, are involved in gastric tumorigenesis via their impact on genetic expression and signaling pathways (Milne et al., 2009). However, the mechanism of GC oncogenesis remains unknown. Nuclear receptor subfamily 2 (NR2F2, group F, member 2, also known as COUP-TFII or ARP1) is a key molecule in mesenchymal-epithelial interaction during organogenesis (Tsai and Tsai, 1997). Meanwhile, Kieback et al. find that NR2F2 is expressed in tumor cell lines of human endometrial (Kieback et al., 1996), pancreatic (Qin et al., 2010), colorectal (Shin et al., 2009), and breast cancers (Prahalad et al., 2010). The expression of NR2F2 in terminally differentiated epithelial cells functions in mesenchymal-endothelial interactions, angiogenesis, tumor growth and metastasis by inhibiting TGF-β-induced growth (Qin et al., 2013).

MicroRNAs (miRs), as small single-stranded noncoding RNAs, are key post-transcriptional negative regulators that completely or partially bind to complementary sites in the 3′-untranslated-region (3′UTR) of target mRNAs. Recent studies show that miRNAs can regulate tumor growth, metastasis and progression (Ma et al., 2007; Ruan et al., 2009; Aleckovic and Kang, 2015). A single miRNA can downregulate the expression of multiple target genes and thereby inhibit tumor metastasis. Therefore, miRNAs can be targeted to modulate the invasion-metastasis cascade (Lin et al., 2015). MiR-27b is a strain of intronic miRNA that regulates chondrosarcoma (Huang et al., 2016), cervical carcinogenesis (Yao et al., 2016) and neuroblastom (Lee et al., 2012).

Interestingly, a recent report demonstrates that the lower level of miR-27b expression correlates with gastric cancer proliferation (Tao et al., 2015). However, little is known about the function of miR-27b in gastric cancer metastasis. In our study, miR-27b was downregulated in gastric cancer tissues, with an inverse correlation with lymph node metastasis. Meanwhile, the miR-27b overexpression inhibited the proliferation and invasion of gastric cancer cells *in vitro* and suppresses tumor growth and liver metastasis of gastric cancer cells *in vivo*. Conversely, miR-27b inhibitors significantly enhanced the proliferation and invasion of gastric cancer cell *in vitro.* Our study concludes that miR-27b plays a suppressive role in gastric cancer metastasis.

## RESULTS

### Survival time shortened by high NR2F2 expression in gastric cancer

**Figure 1:**
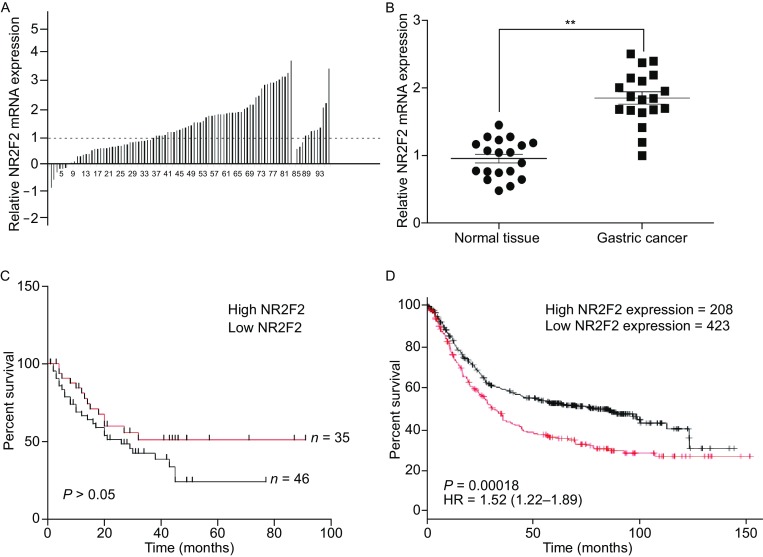
**NR2F2 is up-regulated in human gastric cancer tissues and High NR2F2 level have poor clinical outcome**. (A) The expression of NR2F2 in human gastric cancer tissue samples from oncomine database. (B) qRT-PCR analysis of NR2F2 expression in human gastric cancer tissue samples and their matched normal gastric tissues from 19 gastric cancer patients. (C) The High expression of NR2F2 in human gastric cancer tissues have trends to poor clinical outcome by oncomine database. (D) The High expression of NR2F2 in human gastric cancer tissues have poor clinical outcome by Kalpan meier-Poitter. Scale bars, 100 μm. *P < 0.05, **P < 0.01, ***P < 0.001

The expression of NR2F2 was measured in gastric cancer patients by oncomine database. NR2F2 was significantly up-regulated in gastric cancer tissues compared with normal tissues (Fig. 1A). Q-RT found that NR2F2 expression was significantly higher in gastric cancer tissues than in normal tissues (Fig. 1B). Analysis of immunohistochemical staining and Western blot also support this result (Fig. S1). We subsequently used oncomine database to determine the correlation between the NR2F2 level and the survival time. Although the survival rate had no significant difference (Fig. 1C), the high level of NR2F2 brought a shorter survival time than the low level of NR2F2 did. Then we used Kaplan-meier piotter database to determine the influence of NR2F2 on the survival of gastric cancer patients. The results told that the high level of NR2F2 caused poor clinical survival of patients. (Fig. 1D). All results conclude that the up-regulated NR2F2 level in gastric tissues is negatively correlated with patients’ survival.

### Suppressive role of miR-27b in gastric cancer

**Figure 2:**
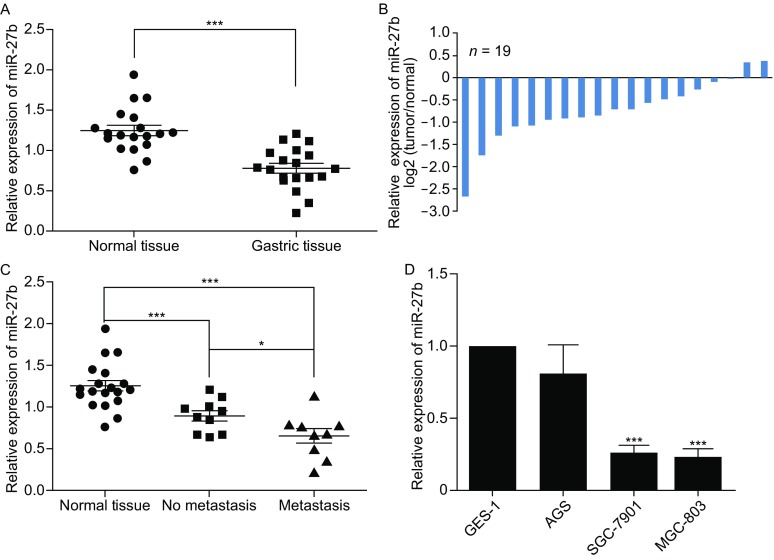
**miR-27b is downregulated in human gastric cancer tissues and metastatic gastric cancer cell lines**. (A) The expression of miR-27b in human gastric cancer tissue samples and matched normal tissues from 19 gastric cancer patients was analyzed by real-time PCR. (B) miR-27b were expressed as log2 fold change to show the relative expression in every paired sample. (C) Correlation between the lymph node metastasis status and the level of miR-27b in gastric cancers (n = 19). (D) The expression of miR-27b in gastric cancer cell lines with different metastatic potentials was analyzed by real-time PCR. Scale bars, 100 μm. *P < 0.05, **P < 0.01, ***P < 0.001

Enough evidence demonstrates that miR-27b, like miRNA, can suppress the proliferation of gastric cancer by targeting ROR1 (Tao et al., 2015). Currently, the function of miR-27b in gastric cancer metastasis remains unclear. To determine its exact function, Q-RT was performed to detect the expression level of miR-27b in gastric cancer tissues and cell lines. As is shown in Fig. 2A, miR-27b expression was significantly decreased in gastric cancer samples compared to matched normal tissues. Of all the patients, 89.47% (17/19) had a lower expression of miR-27b in tumor tissues than that in the adjacent mucosa (Fig. 2B). We further tested the correlation between the level of miR-27b expression and the metastasis of gastric cancer, finding that both were negatively associated (Fig. 2C). We also observed that miR-27b expression was lower in gastric cancer cell lines MGC-803 than in GES-1 cells (human immortalized gastric epithelial cell line) (Fig. 2D). These results prove that the miR-27b level is down-regulated in gastric cancer tissues and gastric cancer cell lines, and is negatively correlated with the metastasis of gastric cancer.

### Cell proliferation, migration and invasion suppressed by miR-27b in gastric cancer

**Figure 3:**
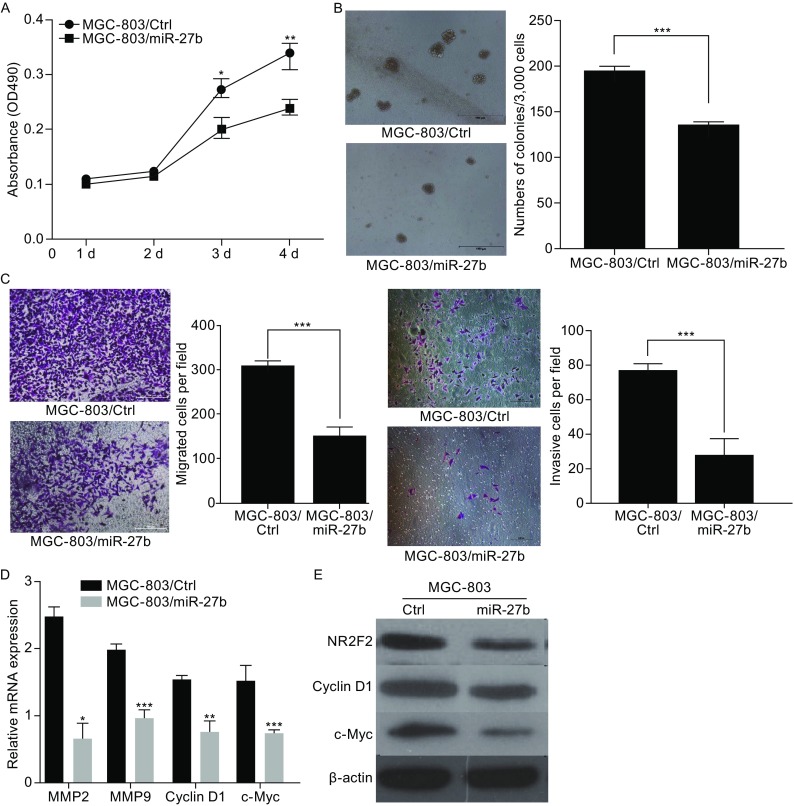
**miR-27b inhibits gastric cancer cell growth, migration, and invasion** ***in vitro***. (A) The effect on cell proliferation of miR-27b overexpression in MGC-803 cells was determined by the MTT assay. (B) Representative images show the colony formation of MGC-803/miR-27b and their control cells (left panel). Average colonies in each well for each group were counted from three independent experiments (right panel). (C) The effects of miR-27b overexpression in MGC-803 cells on cell migration and invasion were analyzed by Transwell migration and Matrigel-coated Transwell invasion analyses. (D) Q-RT analysis of proliferation and metastasis genes. (E) Western blot analysis of proliferation and metastasis genes. *P < 0.05, **P < 0.01, ***P < 0.001

To evaluate the biological function of miR-27b in gastric cancer, the cell line MGC-803 with low expression of endogenous miR-27b was stably transfected with miR-27b by lentiviral infection (Fig. S2A). MTT assays showed that the ectopic overexpression of miR-27b suppressed the proliferation of MGC-803 cells (Fig. 3A). Moreover, soft agar formation assays revealed that overexpression miR-27b could inhibit the expanding of MGC-803 cellular colonies (Fig. 3B). The effects of miR-27b on the migration and invasion of gastric cancer cells were further examined by Transwell assays. The ectopic overexpression of miR-27b in MGC-803 cells dramatically inhibited cell migration (Fig. 3C). Also, we used a Matrigel transmembrane invasion assay to study the invasive properties of MGC-803 cells. Overexpression of miR-27b reduced the invasiveness of gastric cancer cells. Consistent with these phenotypes, then we detected that mRNA levels of MMP2, MMP9, Cyclin D1, c-Myc and the protein levels of c-Myc and Cyclin D1 were downregulated with overexpressed miR-27b (Fig. 3D and 3E). These results suggest that miR-27b can suppress the proliferation, migration and invasion of gastric cancer cells and inhibit the growth and metastasis of gastric cancer.

**Figure 4:**
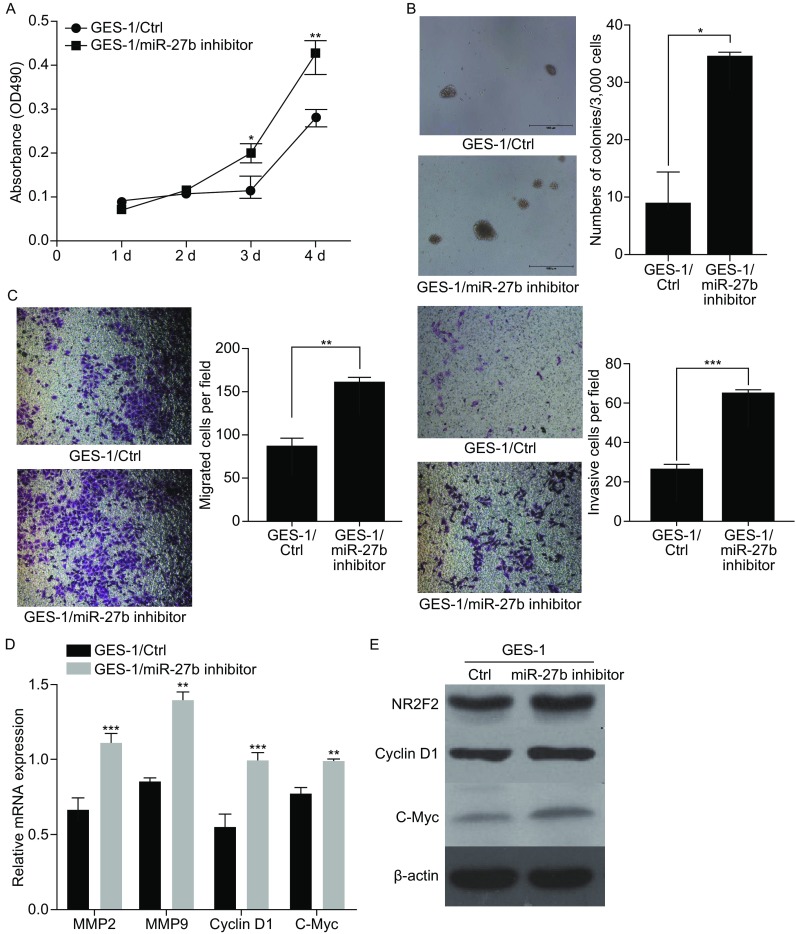
**miR-27b inhibitor promotes gastric cancer cell growth, migration, and invasion** ***in vitro***. (A) The effect on cell proliferation of miR-27b inhibitor in GES-1 cells was determined by the MTT assay. (B) Representative images show the colony formation of GES-1/miR-27b inhibitor and their control cells (left panel). Average colonies in each well for each group were counted from three independent experiments (right panel). (C) The effects of miR-27b inhibitor in GES-1 cells on cell migration and invasion were analyzed by Transwell migration and Matrigel-coated Transwell invasion analyses. (D) Q-RT analysis proliferation and metastasis genes. (E) Western blot analysis of proliferation and metastasis genes. *P < 0.05, **P < 0.01, ***P < 0.001

Proliferation, invasion and metastasis of GES-1 cells promoted by the knockdown of miR-27b Given that the overexpression of miR-27b inhibited the proliferation, migration and invasion of gastric cancer cells, we performed loss-of-function experiments to verify the function of miR-27b in GES-1 cells. First, we used miR-27b inhibitor to knock down endogenous miR-27b in GES-1 cells. Q-RT detected that the level of miR-27b was significantly down-regulated by about 70% after the transfection with miR-27b inhibitor (Supplementary Figure 2B). Next, we explored whether miR-27b inhibitor could augment the proliferation of GES-1 cells. Notably, the miR-27b inhibitor dramatically increased the ability of GES-1 cells in colony formation in a serial passage MTT assay and a soft agar assay (Fig. 4A and 4B). Similar data were obtained by Transwell analysis. The miR-27b increased the migration and invasion of GES-1 cells (Fig. 4C) and the mRNA expression of MMP2, MMP9 and CyclinD1, c-Myc. The protein levels of Cyclin D1 and c-Myc were dramatically upregulated (Fig. 4D and 4E).

### Gastric tumor growth and metastasis inhibited by miR-27b *in vivo*

**Figure 5:**
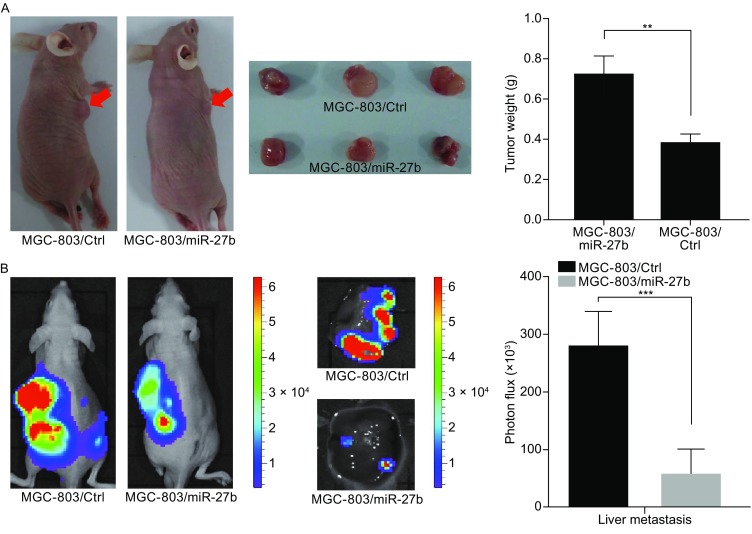
**miR-27b suppresses tumor growth and liver metastasis of gastric cancer cells** ***in vivo***. (A) Representative images of primary tumors in the nude mice orthotopically injected with MGC-803/miR-27b or MGC-803/ctrl cells in the leg (left). Quantification of tumors in each group is shown (right). (B) Representative bioluminescence images of mice injected with MGC-803/miR-27b or MGC-803/ctrl cells into spleen to show liver metastases (left). Quantification of liver metastases was analyzed by bioluminescence measurement (right). Scale bars, 100 μm. *P < 0.05, **P < 0.01, ***P < 0.001

To determine whether miR-27b can inhibit the growth and metastasis of gastric cancer cells in vivo, we generated luciferase-labeled MGC-803/miR-27b cells and their control counterparts, and then injected them into the orthotopic sites or spleens of nude mice. Compared with the mice injected with MGC-803/ctrl cells, the mice injected with a combination of MGC-803 with miR-27b cells displayed significantly smaller and lighter tumors four weeks later (Fig. 5A). Moreover, bioluminescence imaging of mice with a 4-week injection of MGC-803/miR-27b or MGC-803/ctrl cells showed that the metastasis of MGC-803 liver cells was significantly impaired by the ectopic overexpression of miR-27b (Fig. 5B).

### NR2F2 directly targeted by miR-27b

**Figure 6:**
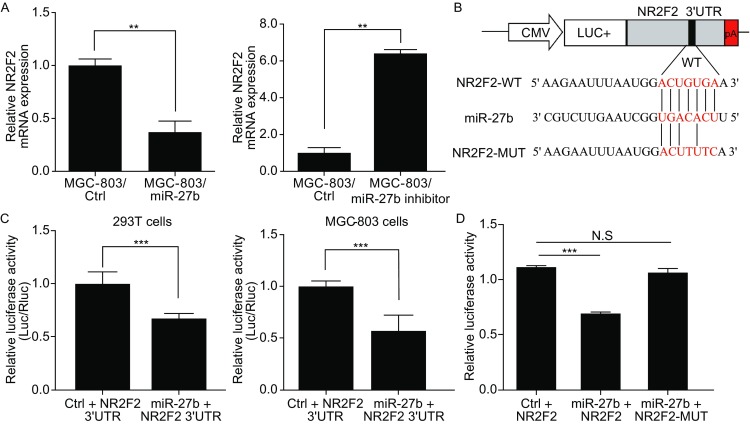
**NR2F2 are direct targets of miR-27b**. (A) The mRNA levels of NR2F2 in MGC-803/miR-27b and MGC-803/ctrl cells were analyzed by real-time PCR. The mRNA levels of NR2F2 in GES-1/miR-27b inhibitor and GES-1/ctrl cells were further analyzed by real-time PCR. (B) Constructed luciferase reporter vectors containing wild-type or mutant 3′UTRs of NR2F2. (C) The effects of miR-27b overexpression on the activity of the 3′UTRs of target genes in 293T cells and MGC-803 cells were analyzed by the dual luciferase reporter assay. (D) The effects of miR-27b expression on the activity of wild-type and mutant 3′UTRs of NR2F2 were analyzed by the dual luciferase reporter assay. N.S.: no significance, *P < 0.05, **P < 0.01, ***P < 0.001

To reveal the underlying mechanism in which miR-27b inhibits the growth and metastasis of gastric cancer cells, we used silico algorithms Targetscan, miRanda and some other methods to predict the target genes of miR-27b. We found NR2F2 with greater than 30% decreased expression upon ectopic miR-27b overexpression in MGC-803 cells and miR-27b inhibitor increased the level of NR2F2 expression (Fig. 6A). We constructed luciferase reporter vectors containing wide-type or mutant 3′UTRs of NR2F2 (Fig.6B). Luciferase activity assays revealed that miR-27b suppressed the expression of luciferase containing 3′UTRs of NR2F2, compared with controls on 293T cells and MGC-803 cells (Fig. 6C). We found possible binding sites of miR-27b in 3′UTRs of NR2F2, and obliterated these sites by QuickChange PCR (Zheng et al., 2004). As is shown in Fig. 6D, the mutation of binding sites in 3′UTRs of NR2F2 reversed the downregulation on luciferase activity induced by miR-27b, and abrogated the suppressing effect of miR-27b overexpression. These results indicate that NR2F2 is the direct target of miR-27b in gastric cancer cells.

## DISCUSSION

Tumorigenesis and metastasis are complex neoplastic processes triggered by a body of transcriptive factors. The abnormal expression of miRNA is involved in tumor proliferation and invasion by inhibiting their target genes (Zhang and Ma, 2012; Zhang et al., 2014b). Therefore, the identification of specific miRNAs and their targets engaged in tumorigenesis and metastasis can provide clues for diagnosing, treating and preventing the cancer. The mechanism that miR-27b participates in gastric cancer proliferation is clear (Tao et al., 2015), but the mechanism in metastasis remains elusive.. In this report, we demonstrated that miR-27b took part in the proliferation and metastasis of gastric cancer. MiR-27b exhibited a decreased expression level in gastric cancer tissues. Moreover, in patients with gastric cancer, a correlation was clarified between lowered miR-27b expression and increased lymphatic metastasis. MiR-27b inhibited proliferation, migration, and invasion of gastric cancer cells *in vitro* and suppressed tumor growth and its metastasis to the liver in vivo. In conclusion, miR-27b is a tumor-suppressing gene in gastric cancer metastasis.

Nuclear receptor subfamily 2 (NR2F2,as known as COUP-TFII) is found in many cancers, including breast cancer (Zhang et al., 2014a), ovarian cancer (Hawkins et al., 2013) and colorectal cancer (Zhou et al., 2016) . Oncomine database and Kaplan meier-piotter defined that NR2F2 was an oncogene in gastric cancer. With Targetscan, miRanda, mirwalk, and Pictar databases, we found highly conserved miR-27b binding sites in the 3′UTRs of NR2F2. Moreover, the miR-27b level was inversely associated with the expression of NR2F2 in gastric cancer tissues, indicating that miR-27b can inhibit gastric cancerous proliferation and metastasis, at least in part, by downregulating the levels of NR2F2.

In all, this experiment provides evidence that miR-27b is a suppressor gene in gastric cancer proliferation and metastasis. MiR-27b can inhibit the proliferation and metastasis of gastric cancer cells by suppressing NR2F2. Furthermore, this experiment provides a potential diagnostic and prognostic marker for gastric cancer metastasis to liver.

## MATERIALS AND METHODS

### Cell lines and clinical samples

GES-1, MGC-803, BGC-823, SGC-7901 and 293T cells were saved in our lab (from Institute of Health Sciences, Shanghai Institute for Biological Sciences, Shanghai). 293T cells were cultured in DMEM supplemented with 10% fetal bovine serum. GES-1, MGC-803, BGC-823 and SGC-7901 cells were maintained in RPMI 1640 media supplemented with 10% fetal bovine serum.

Nineteen paired gastric cancer and normal adjacent tissues were collected from The Affiliated Drum Tower Hospital of Medical School of Nanjing University, Jiangsu, China. All patients were diagnosed pathologically according to the criteria of the American Joint Committee on Cancer by two professional pathologists independently. Specimens were obtained with informed consent and the study was performed in accordance with the approved guidelines by the Medical Ethics Committee of the Affiliated Drum Tower Hospital of Nanjing University (Nanjing, China).

### Plasmid construction and generation of stable cell lines

An hsa-miR-27b-containing flank region was amplified from human genomic DNA and inserted into pCDH-CMV-EF1-GFP+puro (System Biosciences). The entire lengths of the 3’UTRs of NR2F2 were cloned into the pMIR-REPORT miRNA Expression Reporter Vector (Ambion). To generate a miR-27b-expressing stable cell line, a lentivirus-mediated packaging system containing four plasmids, pCDH-miR-27b or control plasmid, pMDL, REV, and VSVG, was used. To knock down miR-27b in GES-1 cells, miR-27b inhibitor was used GenePharma Colone Primer sequences were as follows: miR-27b Fw-GC TCTAGA TTGCCAGGGATTACCACGCAA; Rv-CG GGATCC CTAGCATTCCCAGCAGGAGACAG NR2F2; 3′UTR Fw-CG ACGCGT AAGAAGGGGGAGTGAAACAGAG; Rv-CCC AAGCTT AGCAAGTTGTTCTGACCGACA.

### Western blot

Western blot was performed as described previously (Lin et al., 2015). Cell lysates were separated on SDS-polyacrylamide gels and immunoblot analysis was performed with primary antibodies against NR2F2 (Abcam, Cambridge, MA, USA), β-actin (Millipore, Billerica, MA, USA), c-Myc (Cell Signaling Technology, Danvers, MA) and Cyclin D1 (Sigma-Aldrich).

### Cell growth assay

For the MTT assay, 1000 cells were seeded and transfected in a 96-well plate. After transfection for 1, 2, 3, 4 days, and the absorbance at 490 nm was measured. For soft agar assay, 3000 cells were seeded and transfected in a 6-well plate. After transfection for 14 days, and the size and number of soft agar colonies were measured. Experiments were repeated three times.

### Oncomine and TCGA database analysis

The Oncomine, TCGA and Kalpan meier-plotter database was searched for NR2F2 gene. The data sets containing expression data for NR2F2 were filtered to display up-regulation in gastric cancer versus normal tissue with *P* < 0.05 and have poor clinical outcome.

### Real-time quantitative PCR

Total RNA was extracted from the cultured cells or frozen tissues using TRIzol reagent (Invitrogen, Carlsbad, California, USA). The mRNA was reverse-transcribed by an RT-PCR kit (Invitrogen, Carlsbad, California, USA) according to the manufacturer’s instructions. Quantitative PCR was then performed with primers for miR-27b, NR2F2 using SYBR Green PCR Master Mix (Invitrogen, Carlsbad, California, USA) in a real-time PCR System (Applied Biosystems, Carlsbad, California, USA) following a standard quantitative PCR procedure. Primer sequences used are shown in Table S1.

Relative quantification was performed by normalization to the amount of GAPDH or U6.

### Cell motility and invasion assay

Migration and invasion assays were performed as described previously (Liu et al., 2014). All experiments were performed at least three times in triplicate.

### miRNA reporter luciferase assay

For luciferase reporter assays, 293T cells and MGC-803 cells were seeded into a 24-well plate and co-transfected with 3′UTR-luciferase and either miR-27b or control plasmids. Cells were harvested after two days and assayed using the Dual-Glo Luciferase Assay System (Promega) to determine the relative luciferase activity. The luciferase activity was measured by a luciferin enzyme detection assay kit (Promega) and normalized to Renilla luciferase activity. Each treatment was performed in triplicate in three independent experiments.

### Animal studies

All of the experiments using animals were performed in accordance with a protocol approved by the Animal Care and Use Committee of Nanjing University. For tumor growth assays, 5 × 10^6^ cells were subcutaneously injected into the lower back regions of 6-week-old male nude mice for four weeks (*n* = 3 per group). For orthotropic metastasis, 6-week-old male nude mice were anesthetized and their spleens were exteriorized by laparotomy, and then 5 × 10^5^ cells were injected into the spleens for four weeks (*n* = 3 per group). Tumor growth and hepatic metastases at day 28 were monitored using the live animal Lumina II system (Xenogen IVIS system).

### Statistical analysis

All data were expressed as the mean ± SD. Statistical analysis was performed with Student’s *t*-test. A *P* value less than 0.05 was considered statistically significant.

## Electronic supplementary material

Below is the link to the electronic supplementary material.

Supplementary material 1 (PDF 306 kb)

Supplementary material 2 (PDF 13 kb)
