# Supplementary material for: miR-27b inhibits gastric cancer metastasis by targeting NR2F2
Source: Protein Cell. 2016 Nov 14;8(2):114–22. doi: 10.1007/s13238-016-0340-z (PMC5291775; doi:10.1007/s13238-016-0340-z)
Supplement: Supplementary file 1 — Supplementary material 1 (PDF 306 kb) [file 13238_2016_340_MOESM1_ESM.pdf]

Supplementary Figure 1

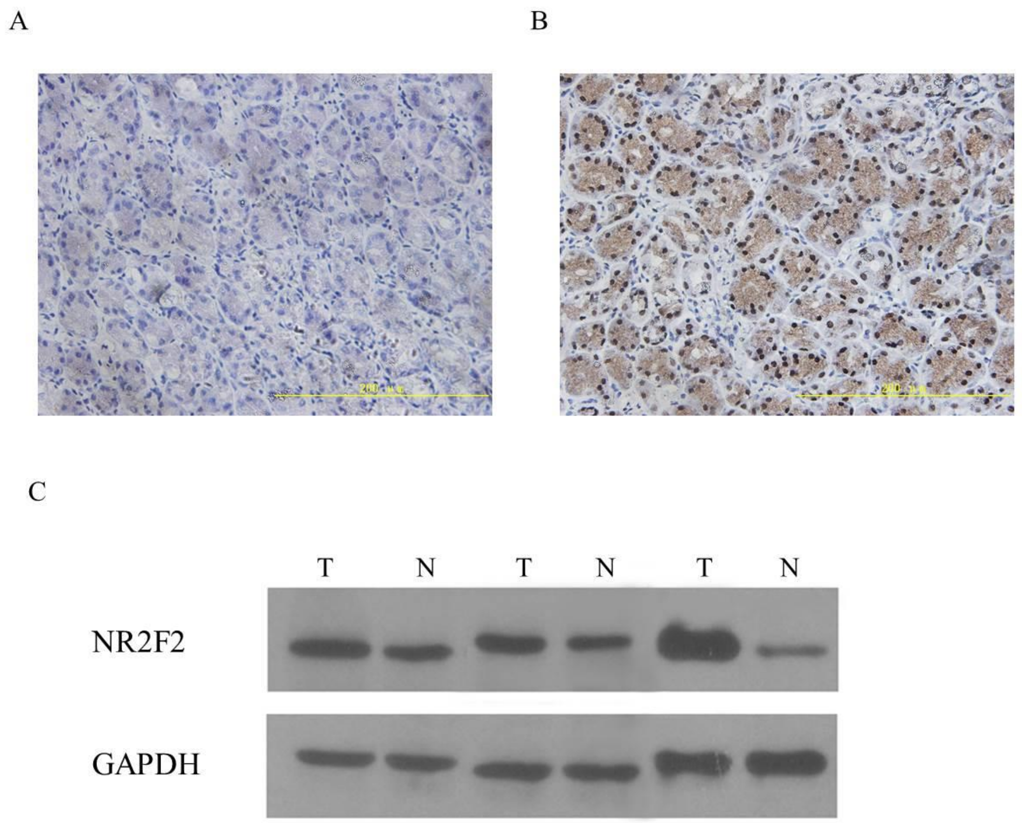

Supplementary Figure 1

Th The expression of NR2F2 in GC cells. (A&B) Representative results of the upregulation of NR2F2 protein in GC specimens by immunohistochemistry（A， Normal Tissue，B，Gastric cancer tissue） (C) The protein levels of ROR1 in six paired GC tissues were detected by western blotting.

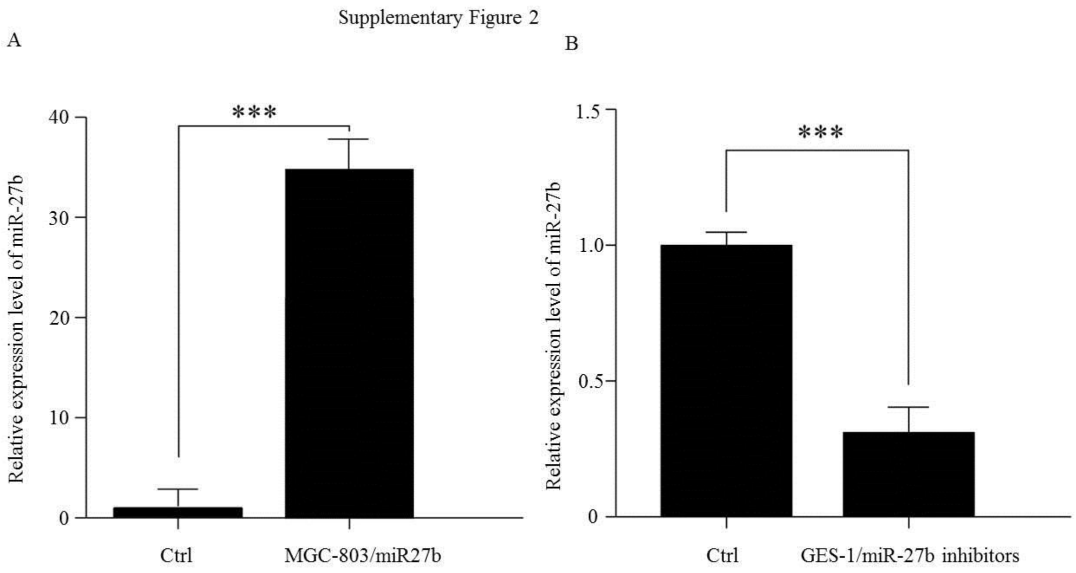

Supplementary Figure 2

Q-RT detects level of miR-27b after transfection with miR-27b / miR-27b inhibitor intro.(A) The miR-27b expression level in MGC-803 cells after ectopic expression of miR-27b.(B) The miR-27b expression level in CES-1 cells after the inhibits efficiency of miR-27b in GES-1 cells were detected by real-time PCR.

*p <0.05, **p <0.01, ***p <0.001

Supplementary Table 1

Primer sequences used in this research.
