# Supplementary material for: miR-27b inhibits gastric cancer metastasis by targeting NR2F2
Source: Protein Cell. 2016 Nov 14;8(2):114–22. doi: 10.1007/s13238-016-0340-z (PMC5291775; doi:10.1007/s13238-016-0340-z)
Supplement: Supplementary file 2 — Supplementary material 2 (PDF 13 kb) [file 13238_2016_340_MOESM2_ESM.pdf]

Supplementary Table 1

NR2F2	Fw<br>Rv	CAAGCACTACGGCCAGTTCAC<br>CACGCTGCGCTTGAAGAAG
MMP2	Fw<br>Rv	GCCCCAGACAGGTGATCTTG<br>GCTTGCGAGGGAAGAAGTTGT
MMP9	Fw<br>Rv	AGACGGGTATCCCTTCGACG<br>AAACCGAGTTGGAACCACGAC
C-MYC	Fw<br>Rv	GCAGCTGCTTACACGCTGGA<br>CGCAGTAGAAATACGGCTGCAC
Cyclin D1	Fw<br>Rv	CCGTCCATGCGGAAGATC<br>ATGGCCAGCGGGAAGAC
GAPDH	Fw<br>Rv	GGTCTCCTCTGACTTCAACA<br>GTGAGGGTCTCTCTCTTCCT
U6-reverse	Reverse primer	CGCTTCACGAATTTGCGTGTCAT
U6	Fw<br>Rv	GCTTCGGCAGCACATATACTAAAAT<br>CGCTTCACGAATTTGCGTGTCAT
